# Current state-of-the-art of device therapy for advanced heart failure

**DOI:** 10.3325/cmj.2014.55.577

**Published:** 2014-12

**Authors:** Lawrence S. Lee, Prem S. Shekar

**Affiliations:** Division of Cardiac Surgery, Brigham and Women’s Hospital, Harvard Medical School, Boston, MA, USA

## Abstract

Heart failure remains one of the most common causes of morbidity and mortality worldwide. The advent of mechanical circulatory support devices has allowed significant improvements in patient survival and quality of life for those with advanced or end-stage heart failure. We provide a general overview of past and current mechanical circulatory support devices encompassing options for both short- and long-term ventricular support.

Heart failure is one of the most common causes of morbidity and mortality in the United States and worldwide. Although transplantation is the gold standard for end-stage heart failure, it is limited by donor supply. In the United States, about 50 000 patients die each year from heart failure but the number of heart transplants remains steady at about 2000 per year (1). Moreover, transplantation is often not optimal or feasible for instances where short-term support may be adequate. While the mainstay of treatment of heart failure has traditionally been medical optimization, non-transplant surgical interventions have grown to play a key role in the care of these patients. Mechanical circulatory support (MCS) options have grown exponentially since the first reports in the mid-twentieth century and are now considered a well-defined and accepted part of heart failure treatment strategies. These surgical procedures comprise an increasingly important part of the armamentarium of the modern cardiac surgeon.

Our intent in this review is to provide a targeted overview of the currently available options for device therapy for heart failure. While the entire spectrum of MCS is quite broad and includes techniques such as intra-aortic balloon pump counterpulsation (IABP), and extracorporeal membrane oxygenation (ECMO), we will focus our discussion on ventricular assist devices (VAD) and total artificial heart (TAH) for the adult population.

## History

John Gibbon reported the first clinical use of MCS when he utilized cardiopulmonary bypass to repair an atrial septal defect in 1953 (1). The first VAD implantation was reported ten years later by Michael DeBakey in a patient with cardiac arrest following aortic valve replacement (2). This patient expired on postoperative day 4. DeBakey reported the first successful use of a VAD for bridge to recovery in 1966 in a patient who received support for 10 days and ultimately was discharged (3). The next several decades were marked by significant technological advancements in device design, spurred in part by initiatives funded by the United States National Heart, Lung, and Blood Institute (NHLBI) of the National Institutes of Health (NIH). 1984 marked the first successful implantation of a TAH, the Jarvik-7-100, by DeVries et al (4). The United States Food and Drug Administration (FDA) gave its first approval in 1994 for an LVAD to be used as a bridge-to-transplant (5).

Since then, continuous advances in device design have led to iterations of VADs that address and decrease complications such as infection, device failure, and thromboembolic events. These newer-generation devices combined with improved surgical techniques have resulted in substantial improvements in clinical outcomes. In 2005, a national registry called the Interagency Registry for Mechanically Assisted Circulatory Support (INTERMACS) was created to serve as a central repository for MCS clinical outcomes data. This prospective registry tracks real-time data points and has proven to be a vital component in understanding aggregate outcomes information for MCS patients.

## Goals of device therapy

The first, and most important, steps when considering MCS therapy are to clearly elucidate the goals of treatment and to expedite early evaluation by a multidisciplinary team. This allows for selection of the appropriate device and timing of intervention for each particular patient. There are five possible goals of MCS: 1. bridge-to-transplant (BTT), 2. destination therapy (DT), 3. bridge-to-recovery, 4. bridge-to-decision, and 5. periprocedural support.

MCS therapy as BTT is utilized in patients deemed to be suitable transplant candidates but needing ventricular support while on the organ waiting list. Although VAD support is widely accepted as standard therapy for these patients, there are no uniform guidelines regarding timing of device placement. Thus, the decision to initiate VAD therapy must consider each individual patient’s operative risk of VAD placement, the estimated waiting time for an available organ, and the estimated mortality while on the waiting list. VAD support in these patients achieves reduction in pulmonary arterial pressures, increase in end-organ perfusion, and improvement from cardiac cachexia, which, in turn, result in the added benefit of improved transplant candidacy. Of the disadvantages and risks of VAD therapy, two are particularly relevant for BTT patients. First, because VAD implantation generally requires a major operation, any subsequent transplantation becomes a re-operation with its attendant risks. Second, exposure to blood products during MCS device implantation can result in sensitization to HLA antibodies, which could potentially make a donor match more difficult.

Patients who are not eligible for heart transplantation can be considered for MCS as DT. These patients are expected to receive MCS therapy for life, with the goals of prolongation of survival or improvement in quality of life. The benefits of MCS support as DT were substantiated in clinical trials (described below), demonstrating significant improvements in survival, functional ability, and quality of life with a DT VAD over optimal medical management.

Some patients experience reverse ventricular remodeling after MCS therapy, ultimately resulting in improvement in ventricular function to such a degree that allows for MCS device explantation. MCS used in these cases is considered bridge-to-recovery. These scenarios often involve biventricular or sequential left followed by right VAD placement. Examples include viral myocarditis and giant cell myocarditis, both of which often resolve with temporary MCS support.

Bridge-to-decision MCS is utilized in settings of acute hemodynamic compromise when there is insufficient time to permit a thorough evaluation of long-term MCS options. Often, the acutely ill patient may have multisystem organ failure and the benefit of long-term MCS is equivocal; in these cases, implantation of short-term MCS as bridge-to-decision can provide support until the patient’s status either improves sufficiently to justify conversion to a long-term device (as BTT or DT) or declines further, obviating the need for additional MCS therapy. Example clinical scenarios can include postcardiotomy shock, acute exacerbation of chronic HF, myocardial infarction, and cardiogenic shock after unsuccessful percutaneous coronary intervention.

Short term MCS can be used as periprocedural support for patients undergoing procedures in the cardiac catheterization laboratory. One example is the use of IABP to augment coronary perfusion during high-risk percutaneous coronary intervention (PCI). More recently, percutaneous LVADs have been used to provide mechanical assistance and may offer superior support when compared to IABP, particularly during hemodynamic depression at the time of balloon inflation in PCI. Percutaneous LVADs can be placed prophylactically before high-risk PCI or as rescue therapy in setting of periprocedural emergencies such as coronary dissection or cardiogenic shock. These MCS devices can be removed after completion of the procedure or left in place as a bridge to a definitive cardiac surgical operation.

It is important to note that these classifications are not fixed. A patient may receive MCS under one classification but changes in clinical status may modify that patient’s classification. For instance, a patient may receive an LVAD as BTT and then recover sufficient ventricular function such that the LVAD can be explanted, thus classifying MCS support as bridge-to-recovery. Similarly, a patient with a short-term MCS device as bridge-to-decision who undergoes heart transplantation could then be considered as having had MCS as BTT.

## Device options

MCS devices can be classified and categorized by four factors: duration of support (short-term vs long-term), configuration of ventricular assist (biventricular vs univentricular), pump flow pattern (pulsatile vs continuous-flow), and location of implantation (extracorporeal vs intracorporeal).

### Short term device options

MCS is considered short-term when the duration of support is on the order of days to weeks. Currently available devices are listed in Table 1. The first short-term MCS device to receive FDA approval was the Abiomed BVS5000 (ABIOMED Inc, Danvers, MA, USA). This device is a pulsatile, pneumatically-driven pump with a large external controller. In 1993, a multicenter, non-randomized clinical trial showed that in patients with postcardiotomy shock, implantation of the Abiomed BVS5000 resulted in 55% of patients weaning from support with 29% surviving to discharge (6).

**Table 1:** Short-term mechanical circulatory support (MCS) devices

Device	Manufacturer	Mechanism	Assist	Location
Intra-aortic balloon pump counterpulsation	Multiple	Counterpulsation	Not applicable	Not applicable
Extracorporeal membrane oxygenation	Multiple	Cardiopulmonary bypass	Not applicable	Not applicable
BVS5000	ABIOMED	Pneumatic, pulsatile	Right, left, or bi-ventricular	Extracorporeal
CentriMag	Thoratec	Electric, centrifugal continuous	Right, left, or bi-ventricular	Extracorporeal
Impella	CardiacAssist	Electric, axial continuous	Percutaneous; left	Intracorporeal
TandemHeart	ABIOMED	Electric, centrifugal continuous	Percutaneous; right, left, or bi-ventricular	Extracorporeal

A recent addition to the armamentarium of short-term MCS options is the CentriMag (Thoratec Corp, Pleasanton, CA, USA). This device is an extracorporeal, continuous centrifugal-flow pump with a magnetically levitated rotor and external controller. The CentriMag is capable of uni- or bi- ventricular support with flows up to 10 L/min (7,8). One of the key advantages of this device is its portability and versatility. The CentriMag has been used to provide support for a period of days to weeks as a bridge-to-decision as well as bridge-to-transplant device (9,10). Frequently, the CentriMag is utilized for short-term right ventricular support in patients with long-term LVADs who demonstrate initial right heart dysfunction (11-13).

Two percutaneous devices are currently available for short-term MCS. The TandemHeart (CardiacAssist Inc, Pittsburgh, PA, USA) is a continuous-flow centrifugal pump with an external controller capable of flow rates up to 5 L/min. This device is placed in the cardiac catheterization laboratory with transseptal left atrial inflow via percutaneous femoral venous access and outflow through contralateral femoral arterial access. Though initially designed to provide temporary support for patients undergoing high-risk percutaneous cardiac interventions, the TandemHeart has also proven its utility in postcardiotomy heart failure and cardiogenic shock. Several studies have shown improvements in hemodynamic parameters and cardiac indices with TandemHeart support in bridge-to-recovery and bridge-to-decision settings (14,15). The TandemHeart can be explanted either at the bedside when support is no longer needed or in the operating room at the time of transplantation or implantation of a longer-term MCS device.

The Abiomed Impella (ABIOMED Inc) is another percutaneous short-term MCS option. This device is a continuous-flow, axial pump with an external controller. There are two models, the Impella 2.5 and the Impella 5.0, with the number designating the maximal flow rate delivered: the Impella 2.5 can deliver up to 2.5 L/min and the 5.0 up to 5.0 L/min. The device is designed to rest across the aortic valve and pump blood from the left ventricle directly into the ascending aorta. The Impella 2.5 can be inserted percutaneously via the femoral vessels in the cardiac catheterization laboratory. In a randomized clinical trial comparing the Impella to IABP counterpulsation, the Impella demonstrated superior hemodynamic support but 30-day mortality was similar in the two groups (16). One limitation of the Impella 2.5 is that the maximal flow rate of 2.5 L/min may be inadequate for larger patients or scenarios that require more flow. The Impella 5.0 addresses this shortcoming by allowing for greater flow rates. However, the 5.0 is a larger device which requires surgical cut-down for placement through a peripheral vessel.

The obvious advantage of percutaneous devices is implantation performed without the need for surgery. Moreover, the insertion of percutaneous devices generally is technically less difficult and thus can be performed more expeditiously, which can prove extraordinarily beneficial in the acute setting.

### Long term device options

The early design of long-term MCS devices featured pulsatile pump technology because pulsatility was believed to be necessary for organ perfusion and recovery. These early devices contained valves to allow for unidirectional blood flow and ventricular sacs that could produce a stroke volume of 65-85 mL. Because of the complexity and number of mechanical parts, physical wear and tear of the device became a limiting factor in device durability (17-19). Subsequent generations of these devices featured continuous flow pumps with fewer moving parts and improved durability. Studies demonstrated that continuous flow devices provided improved outcomes with regards to survival, quality of life, functional capacity, and adverse events (20-25). Table 2 lists currently available long-term MCS devices.

**Table 2:** United States Food and Drug Administration (FDA) approved and investigational long-term mechanical circulatory support devices

Device	Manufacturer	Mechanism	Assist	Location
Novacor*	World Heart	Pneumatic, pulsatile	Left	Intracorporeal
HeartMate XVE*	Thoratec	Electric, pulsatile	Left	Intracorporeal
AB5000	ABIOMED	Pneumatic, pulsatile	Right, left, or bi-ventricular	Paracorporeal
Paracorporeal Ventricular Assist Device	Thoratec	Pneumatic, pulsatile	Right, left, or bi-ventricular	Paracorporeal
Implantable Ventricular Assist Device	Thoratec	Pneumatic, pulsatile	Right, left, or bi-ventricular	Intracorporeal
HeartMate II	Thoratec	Electric, axial continuous	Left	Intracorporeal
HeartWare LVAS	HeartWare	Electric, centrifugal continuous	Left	Intracorporeal
Jarvik 2000 FlowMaker	Jarvik Heart	Electric, axial continuous	Left	Intracorporeal
HeartAssist 5	MicroMed	Electric, axial continuous	Left	Intracorporeal
DuraHeart	Terumo	Electric, centrifugal continuous	Left	Intracorporeal
CardioWest TAH	Syncardia	Pneumatic, pulsatile	Not applicable	Intracorporeal
Abiomed TAH	ABIOMED	Electrohydraulic, pulsatile	Not applicable	Intracorporeal

*No longer commercially available in the United States.

### First generation VADs

The Thoratec HeartMate XVE (Thoratec Corp) and Novacor LVAD system (WorldHeart Corp, Oakland, CA, USA) were among the earliest implantable VADs. The HeartMate XVE quickly became the first widely utilized LVAD worldwide. It is powered by an electrically driven pulsatile pump capable of generating up to 10 L/min of flow. It contains porcine valves in the inflow and outflow conduits to maintain unidirectional flow. The inflow cannula is attached to the LV apex, the outflow cannula to the ascending aorta, and the device itself is implanted behind the rectus sheath in the subcostal region or in the peritoneal cavity. The size of the device requires that the patient’s body surface area (BSA) be greater than 1.8 m^2^, thus excluding most children and small adults. The pump receives power from an external power source via a driveline that enters the body through the right side of the abdomen. The HeartMate XVE is constructed with textured blood-contacting surfaces that become covered by a “pseudoneointimal” layer, eliminating the need for systemic anticoagulation. The HeartMate XVE is the only pump to date with this property, thus making this device particularly attractive for patients with a contraindication to systemic anticoagulation (17).

The HeartMate XVE was the first VAD to demonstrate MCS as a viable option for use as DT. In 2001, the Randomized Evaluation of Mechanical Assistance for the Treatment of Congestive Heart Failure (REMATCH) trial randomized 129 patients with end-stage heart failure who were not candidates for cardiac transplantation to receive either optimal medical management or implantation of the HeartMate XVE (17). Survival at one and two years was 52% and 23%, respectively, in the LVAD group vs 25% and 8% in the medical management cohort. In patients under the age of 60, the one-year survival was 74% with the LVAD compared to 33% with medical management. The most common causes of death in the LVAD group were infection (41%) and device failure (17%). Quality of life and functional capacity were also markedly improved in the LVAD cohort compared to the control patients. The results of this landmark trial led to FDA approval of the HeartMate XVE as the first VAD for DT. A follow-up study in patients with the HeartMate XVE, however, showed that the need for device exchange due to malfunction or failure approached nearly 73% at two years, thus reinforcing the need for improved device design (26). While first generation LVADs helped to usher in the era of long-term MCS, they are rarely implanted today as studies have shown superior outcomes with newer generation devices (17).

Other examples of first generation devices include the Abiomed AB5000, the Thoratec Paracorporeal Ventricular Assist Device (PVAD) II (Thoratec Corp), and the Thoratec Implantable Ventricular Assist Device (IVAD). All are pulsatile pumps capable of bi- or uni- ventricular support. The Thoratec PVAD has been in clinical use for over 20 years and has the additional benefit of allowing patients to be discharged home with the device in place. In one series, 47% of patients supported with a PVAD survived to discharge while 68% underwent heart transplantation (27). The Thoratec IVAD is based on the PVAD but is smaller and completely implantable, thus permitting more mobility and independence after discharge. A portable driver interface allows device evaluation and control by patients or their caregivers, and patients report an increase in their quality of life with this type of system (28).

### Second generation VADs

The Thoratec HeartMate II (HMII) is a second generation LVAD that received FDA approval in 2008 for BTT use. It is the most widely implanted LVAD to date with over 10 000 patients worldwide. The HMII is composed of an electrically powered rotary continuous flow pump, where the only moving part is the axial rotor. This imparts tangential velocity and kinetic energy to the blood, resulting in generation of a net pressure rise across the pump. Power is supplied by an external source that connects via a percutaneous driveline usually from the right side of the abdomen. The pump can generate flow rates of up to 10 L/min and is preload dependent and afterload sensitive. Like the HeartMate XVE, the HMII has the inflow cannula connected to the LV apex and the outflow cannula to the ascending aorta. The HMII is also implanted in a preperitoneal pocket in the left subcostal space, but its smaller size allows it to be used in smaller patients (BSA can be as low as 1.2 m^2^) than the XVE. As with all continuous flow devices, patients with the HMII do not have a palpable pulse. Some limitations of this type of device are hemolysis, ventricular suction events, and thrombus formation with pump stoppage (29,30).

In 2007, the results of a multicenter trial investigating the HMII as BTT were published in the *New England Journal of Medicine* (29). This trial involved 26 centers enrolling 133 patients with NYHA Class IV heart failure who were all on the active wait list for heart transplantation. All patients underwent HMII implantation. At the end of six months, 75% of patients had either survived to transplant, had recovered sufficiently to survive explant of the HMII, or were still alive and awaiting transplant. There were no device failures, and functional capacity and quality of life also improved significantly. Complications included bleeding necessitating surgery (31%), device-related infection (14%), stroke (8%), and pump thrombosis (1.5%) (29). A separate multicenter trial of 281 patients with the HMII showed six and twelve month survival of 82% and 73%, respectively (30). Furthermore, functional status had recovered markedly by six months such that 83% of patients improved to NYHA class I or II. Pump replacement was required in 4% of patients (29,30).

The first report of post-FDA approval HMII outcomes was published in 2011 (31). This was a prospective study of the first 169 patients who received the HMII after it had become commercially available. This group was compared to an INTERMACS cohort of 169 patients receiving another commercially available LVAD for BTT (135 with the Thoratec HeartMate XVE and 34 with the Thoratec IVAD, both pulsatile devices). At six months, 90% of the HMII patients had either survived to transplant, recovered sufficiently to survive explant of the device, or were still alive and awaiting transplant vs 80% of the pulsatile pump group. Overall twelve month survival was 85% in the HMII group and 70% in the comparison cohort, and 92% of the HMII patients were discharged home vs 75% of the pulsatile pump patients. This study again confirmed the superiority of continuous-flow VADs over pulsatile devices as BTT.

In a separate trial investigating the HMII as DT support, 200 patients at 38 different centers were randomized 2:1 to receive the HMII or the HeartMate XVE (32). These patients were not eligible to receive a heart transplant, had an LVEF of less than 25%, were NYHA class IIIb or IV, and were IABP-dependent for 7 days or inotrope-dependent for 14 days. Ultimately, 133 patients received the HMII and 59 received the XVE. The primary endpoint was freedom from disabling stroke and freedom from reoperation for device malfunction at the end of two years. 46% of HMII patients reached the primary endpoint vs 11% of the XVE patients. Stroke occurred in 11% in the HMII group compared to 36% in the XVE group, and reoperation for device replacement was required in 10% of the HMII group vs 12% of the XVE group. While the rates of reoperation for pump replacement were similar, the causes of device malfunction were notably different: bearing wear and valve degeneration or infection were culprits in the XVE, while broken percutaneous leads was most common in the HMII. One- and two-year actuarial survival in the HMII group was 68% and 58%, respectively, vs 55% and 24% in the XVE group. This pivotal trial demonstrated superior outcomes and durability with the continuous-flow HMII over the pulsatile flow XVE for DT support.

In 2011, VAD centers began to report a perceived increase in the frequency of HMII pump exchanges due to thrombus, thus prompting an analysis of the INTERMACS database. A review of 6910 patients from 132 institutions who received an HMII between 2008 and 2012 revealed an overall incidence of pump thrombus of 5.5% (33). There was a statistically significant increase in pump exchange or death due to pump thrombus during 2011 and 2012: the freedom from pump exchange or death due to thrombus decreased from 99% at 6 months before 2011 to 96% in 2011 and 94% in 2012. Overall survival of 80% at 1 year and 70% at 2 years, however, remained unchanged regardless of year of device implantation. No clear device-related or implantation technique-related etiology was identified as the cause of the increased pump thrombosis. Rather, risk factor analysis suggested a number of patient-related factors that may contribute to the risk of pump thrombosis, and vigilant monitoring of anticoagulation parameters, thrombosis risk, hemolysis, infection, and mechanical failure was recommended.

Other second generation LVADs include the HeartAssist 5 (MicroMed Cardiovascular Inc, Houston, TX, USA), Jarvik 2000 FlowMaker, and the HeartWare (HeartWare International Inc, Framingham, MA, USA) left ventricular assist system (LVAS). The HeartAssist 5 is a small version of the Micromed DeBakey pump that can be implanted within the pericardial space. The Jarvik 2000 FlowMaker is also implanted in the pericardial space directly into the LV apex with an outflow attachment to the ascending or descending aorta. This portable device is capable of providing partial support with flow rates up to 7 L/min and allows the patient to manually adjust the pump speed depending on activity level. One key advantage of the Jarvik 2000 is its small size: it is about the size of a C battery, significantly smaller than the Thoratec HMII. The Jarvik 2000 is currently in clinical trials and awaiting FDA approval.

The HeartWare LVAS has a unique design that combines magnetic levitation and hydrodynamic suspension that eliminates any contact between the impellar and pump housing. It is implanted intrapericardially with LV apical inflow and outflow to the ascending aorta. The HeartWare LVAS operates at a fixed pump speed and is capable of producing flow rates of 10 L/min. This device was studied in a BTT trial (ADVANCE) in the United States from 2008 to 2010 (34). 140 patients with the HeartWare LVAS were compared to 499 patient controls from the INTERMACS registry who received a different LVAD as BTT. At six months, 92% of the HeartWare patients survived to transplant, recovered, and survived device explant, or were alive and still awaiting transplant compared to 90.1% of control patients. Six- and twelve-month survival was 94% and 90.6%, respectively, in the HeartWare group and 90.2% and 85.7% in the comparison group. The HeartWare LVAS received FDA approval for BTT in 2012 and is currently undergoing trials for DT use.

### Third generation VADs

Third generation of devices are centrifugal continuous-flow pumps with an impeller or rotor suspended in the blood flow path using either magnetic or hydrodynamic levitation. This eliminates component-to-component contact, thus reducing frictional wear and heat generation and theoretically increasing device durability and reliability. The magnetic levitation systems are one of three types: external motor-driven, direct-drive motor-driven, or bearingless motor. Third-generation LVADs are more widely used in Europe, where they reportedly comprise nearly 50% market share (25). Initial reports indicate non-inferiority of these third-generation LVADs when compared to second-generation devices (35).

Examples of third generation devices include the Terumo DuraHeart (Terumo Heart Inc, Ann Arbor, MI, USA) and Thoratec HeartMate III. The Terumo DuraHeart is commercially available in Europe and is undergoing clinical trials in the United States. The device is a centrifugal continuous flow pump that is implanted in a preperitoneal pocket and is capable of flow rates up to 8 L/min. Six and twelve month survival in a European BTT trial was 81% and 77%, respectively, with no pump failure or thrombosis during an average support duration of eight months. The most common adverse events were neurological complications (27%), right heart failure (27%), and infection (18%) (36). The Thoratec HeartMate III is a centrifugal pump, which is in clinical trials. It remains to be seen whether these third generation devices provide substantial improvement in outcomes and adverse events over currently available second generation continuous flow VADs.

### Total artificial heart

The quest for a TAH began with the advent of MCS devices. Conceptually, the TAH is quite attractive as it offers the ability to replace the entire failing heart. The successful design and implementation of a TAH in clinical practice, however, has been much more elusive. Furthermore, the rapid expansion of VAD technology has overshadowed TAH development. As a result, the majority of patients with advanced heart failure can be successfully managed with an LVAD or BIVAD and the necessity of a TAH is debatable. The current indications for a TAH are limited and include severe bi-ventricular failure, myocardial rupture, post-transplant rejection, LV thrombus, restrictive cardiomyopathy (amyloidosis and hypertrophic cardiomyopathy), and refractory ventricular dysrhythmia.

At present, the only TAH that is FDA approved is the CardioWest TAH (Syncardia Systems Inc, Tucson, AZ, USA), a pneumatically powered pulsatile dual chamber device. Two percutaneous drivelines connect the TAH to an external power source. The patient must remain in hospital, although the recent development of a portable wearable power source, the Freedom Driver, may ultimately permit discharge to home. The Freedom Driver allows considerably greater mobility and is currently undergoing trials. The design of the CardioWest device consists of four prosthetic tilting disk valves and two 70 cc pumping chambers that can produce flow rates up to 9.5 L/min. The relatively large size of the device requires a minimal body surface area of 1.7 m^2^ (37,38). There have been nearly 1000 implants worldwide. A multicenter BTT trial compared 81 critically ill patients with biventricular failure who received the CardioWest TAH to a 35-patient historical control group who received medical therapy alone (39). Survival to transplantation was 79% in the CardioWest TAH cohort and 46% in the control group. Post-transplantation survival was also superior in the TAH group, with 86% at one year and 64% at five years vs 69% and 34% in the control patients. The CardioWest patients also showed substantial improvement in hepatic and renal function parameters within weeks of TAH implantation. Adverse events included significant bleeding (28%), driveline infection (21%), stroke with residual neurologic deficit (0.07%), and device malfunction (0.01%).

The Abiomed TAH (ABIOMED Inc) is available in the United States under an FDA humanitarian device exemption. This TAH is a completely implantable titanium and plastic dual chamber pump utilizing an electrohydraulic mechanism to alternate ejection between the two chambers. Because the entire device is intracorporeal, there is no external driveline. Implantation of the Abiomed TAH began in 2001 under an FDA investigational device exemption. A total of 14 patients received the device with the longest duration of support being 512 days (40).

## Conclusions

There have been remarkable advancements in MCS options since the first days of cardiopulmonary support. Landmark trials such as REMATCH firmly established MCS as a viable, effective treatment strategy for advanced heart failure. With MCS, patients with end stage heart disease, regardless of transplant eligibility, are now living longer than ever with improved quality of life. Collaborative database registries such as INTERMACS provide contemporary, real-time data to assist in the optimization of risk stratification and patient selection. Evolution of device design will hopefully continue to decrease complications and result in smaller, portable, and more durable device options.
